# Multi objective reinforcement learning driven task offloading algorithm for satellite edge computing networks

**DOI:** 10.1038/s41598-025-10553-6

**Published:** 2025-07-05

**Authors:** Sai Xu, Jun Liu, Jiawei Tang, Xiangjun Liu, Zhi Li

**Affiliations:** 1 School of Computer Science and Engineering, Northeastern University, Shenyang, 110169 China; 2 Neusoft Research, Shenyang, 110179 China; 3 CS&S Information System Engineering Co.,Ltd, Beijing, 100081 China; 4 School of Information Science and Engineering, Shenyang Ligong University, Shenyang, China

**Keywords:** Space-based information network, Satellite edge computing, Task offloading, Reinforcement learning, DQN, Engineering, Aerospace engineering

## Abstract

Satellite edge computing (SEC) has become a revolutionary paradigm to improve the quality of service, reduce the pressure on satellite-terrestrial backhaul bandwidth and reduce the average response delay of task requests. In this paper, we propose a task offloading algorithm based on K-D3QN to meet the rapidly growing demand of ground users. This algorithm improves the DQN algorithm by incorporating a satellite resource clustering module, a DDQN algorithm, and a competitive network mechanism module. The offloading decision-making process comprehensively considers three optimization objectives: task latency, resource utilization, and load-balancing degree, to achieve dynamic multi-objective optimization. Experimental results shown that the algorithm significantly reduces task latency, improves resource utilization and load-balancing degree.

## Introduction

The Space-Based Information Network (SIN)^1,2^ is a system that uses a network of satellites deployed in space to transmit information. This network relies on communication links between satellites to transfer data, voice, video, and other forms of information from senders on Earth to recipients. SIN is characterized by its global coverage, high-speed transmission, and wide-ranging applications, which enable communication and information exchange in various domains. It is widely used in fields such as military operations, communications, meteorology, navigation, and scientific research. During natural disasters or emergency situations, SIN can provide timely communication and data transmission capabilities, assisting organizations and individuals in coordinating actions and obtaining the necessary information^3^.

In the traditional satellite information processing model, data transmission and data processing are treated as entirely separate processes. Satellites collect data and transmit it to a ground-based cloud center for processing via satellite-terrestrial links. The cloud center then returns the computation results to the satellites or other ground users^4,5^. However, with the rapid advances in space science and technology, satellite edge computing (SEC) has emerged as a novel paradigm. SEC leverages low Earth orbit (LEO) satellites as edge servers to provide computational services, thus enhancing processing capabilities^6–9^. Despite its promising potential, the SEC faces several challenges, including limited satellite resources, dynamic characteristics of network topology, and network security^10–14^. The challenges encountered in mobile edge computing (MEC) are similar in SEC^15–17^.

To optimize application Quality of Service (QoS), SEC architecture and task offloading have emerged as critical research frontiers in SIN^18,19^. Dong et al.^20^ investigated the computational offloading problem in LEO satellite networks. They formulated a joint optimization problem to minimize energy consumption. Tong et al.^21^ design a MEC-enabled STN architecture that achieves efficient offloading and resource allocation of computational tasks for IoT devices through interstellar collaboration. This is accomplished using a task offloading decision algorithm based on the Gray Wolf Optimizer algorithm and a computational resource allocation algorithm based on the Lagrange multiplier method, significantly reducing task completion latency. Zhang et al.^22^ proposed a multiobjective optimization model that trades off between energy consumption, cost, and response time, and a Petrinet-based constraint amending method with polynomial complexity and generated offloading results satisfying constraints. Wei et al.^23^ proposed a multi-agent Q-learning algorithm, including multi-group dual-agent Q-learning, based on local state observation and global reward calculation. The convergence of the proposed multi-agent Q-learning algorithm is also theoretically analyzed. This algorithm can effectively reduce offload latency. Liu et al.^24^ proposed a computation offloading decision scheme based on Markov and deep Q networks (DQN), which can effectively reduce latency and energy consumption during task computation offloading in the space-air-ground integrated network environment. In^25–27^, the authors have conducted extensive research on network security, obtaining groundbreaking results that contribute significantly to the field. Wu et al.^28^ consider LEO satellite mobility and dynamic load levels to optimize the computation offloading strategy in satellite edge computing. Wei et al.^29^ analyzed a hybrid cloud and edge computing architecture for LEO satellite networks. It constructs a joint optimization problem for offloading decisions and computational resource allocation, with the aim of reducing user processing latency and energy consumption despite the limited computational resources at edge nodes.

Unfortunately, most related studies focus primarily on energy consumption or response latency, without fully addressing the dynamic changes in satellite network topology and the impact of task priority on offloading efficiency. Motivated by the above, we propose the K-D3QN offloading strategy, which comprehensively considers factors such as limited resources of SEC, dynamic topology, and task priority.

The main contributions of this paper can be summarized as follows.

1. The system model of task offloading in satellite edge network scenarios is established. The task offloading decision is formulated as a multi-objective optimization problem, aiming to optimize system resource utilization, task response latency, and system load-balancing degree.
2. We propose a task offloading algorithm, K-D3QN, for satellite edge computing. First, the K-means^30^ algorithm is utilized to cluster satellite resources based on their capabilities, effectively reducing the complexity of the problem-solving space. Next, a parameter update mechanism is introduced to mitigate the overestimation of Q-values in the traditional Deep Q Network(DQN)^31^ algorithm. Finally, a competitive network mechanism is incorporated to accelerate the algorithm’s convergence.
3. We completed the simulation and results analysis. The results show that our proposed task offloading strategy has better results in terms of offloading success rate, average completion time, and load-balancing degree.

The rest of the paper is organized as follows: Section 2 describes the system model. Section 3 introduces the K-D3QN algorithm. Section 4 presents the experimental setup and results analysis, and Section 5 concludes the entire work.

## System model

**Fig. 1:**
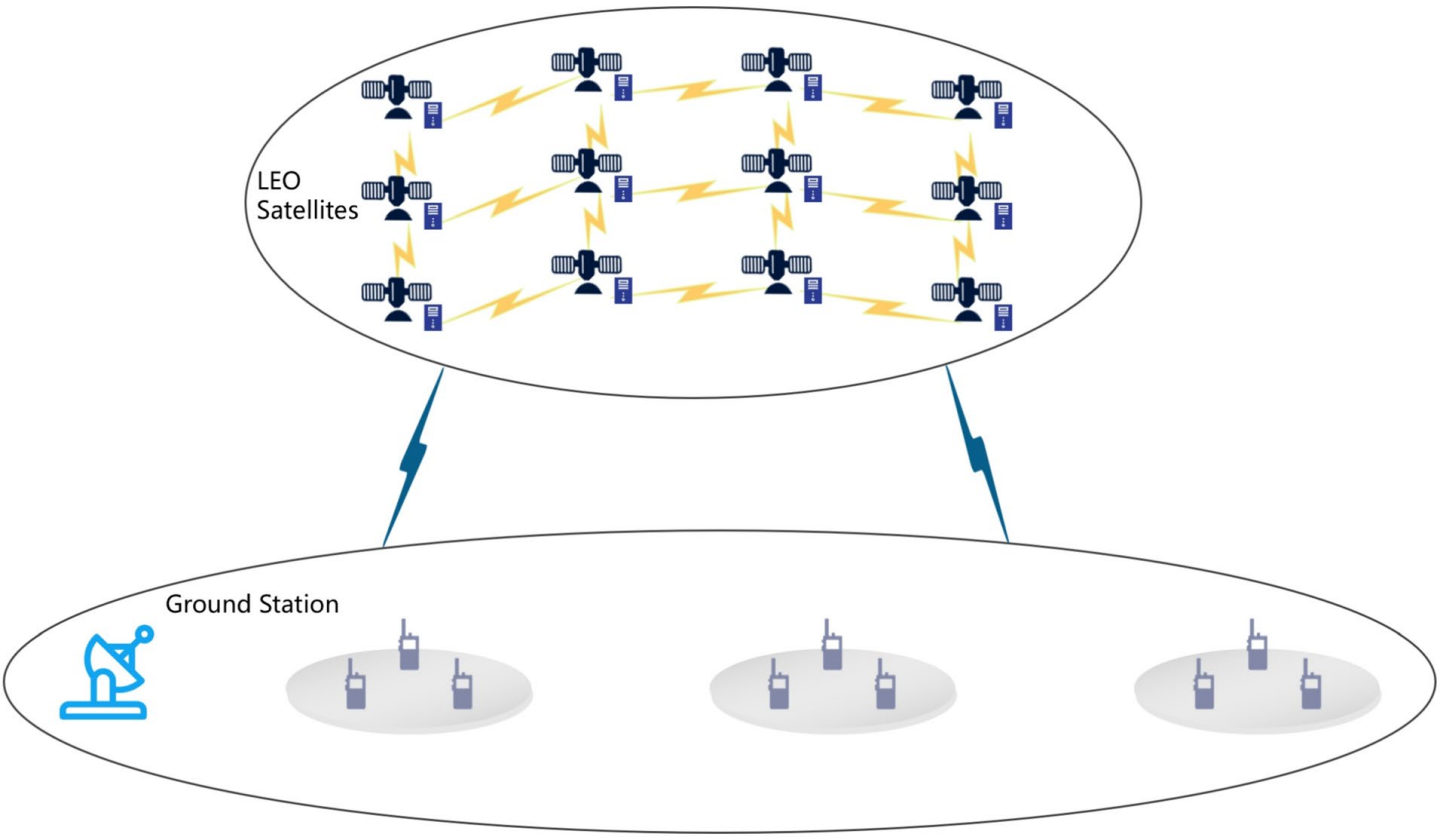
Satellite edge computing network architecture.

As shown in Fig. 1, the system consists of *n* LEO satellites and *m* ground terminals. These ground terminals send requests to the satellite network via a ground station, where each LEO satellite acts as an edge server to provide on-orbit computing, storage, and communication services.

The main notations used in this paper are summarized in Table 1.

**Table 1:** List of main notations.

Notation	Description
*G*	Satellite network topology
*V*	Collection of LEO satellites
*E*	Collection of satellite links
*n*	Number of LEO satellites
*m*	Number of terminals
$f_c$	CPU frequency of satellite
$R_c$	Computing capacity of satellite
$R_s$	Storage capacity of satellite
$R_L$	Connections between satellites
*P*	Priority of task
*Data*	The data size of tasks
$T_C$	The computational resources required for the task
$T_S$	The storage resources required for the task
$T_L$	The link resources required for the task
$T_{start}$	The start time for the task
$T_{dl}$	The maximum latency for the task
*g*	Weight for optimization target

### Satellite network topology model

The network topology model represents satellites as nodes and inter-satellite links as edges^32^. The positions and connections of satellite nodes change periodically. In this paper, the satellite network topology can be characterized as shown in Equation (1).

1 $$\begin{aligned} G=\left( V,E,W_V\left( t\right) ,W_E\left( t\right) \right) . \end{aligned}$$

Where $V=(V_1,V_2,\cdots ,V_n)$ represents the set of satellite nodes, and $E=(e_{11},e_{12}, \cdots , e_{nn})$ characterizes the links, denoting the connectivity between all satellites. $W_V(t)=\left\{ w_{v_i}\left( t\right) | v_i\in V\right\}$ denotes the weight attributes of the nodes at moment *t*, such as resource type and resource capacity. Similarly, $W_E(t)=\left\{ w_{e_{ij}}\left( t\right) | e_{ij}\in E\right\}$ represents the weight attributes of the links at time *t*. The connection states of inter-satellite links and their weights vary over time and can be effectively represented by a dynamic adjacency matrix:

2 $$\begin{aligned} W_E\left( t\right) =\left[ \begin{matrix}e_{11}\left( t\right) & \cdots & e_{1n}\left( t\right) \\ \vdots & \ddots & \vdots \\ e_{n1}\left( t\right) & \cdots & e_{nn}\left( t\right) \\ \end{matrix}\right] . \end{aligned}$$

Where $e_{ij}\left( t\right)$ represents the link resources between satellite node *i* and satellite node *j* at time *t*.

**Fig. 2:**
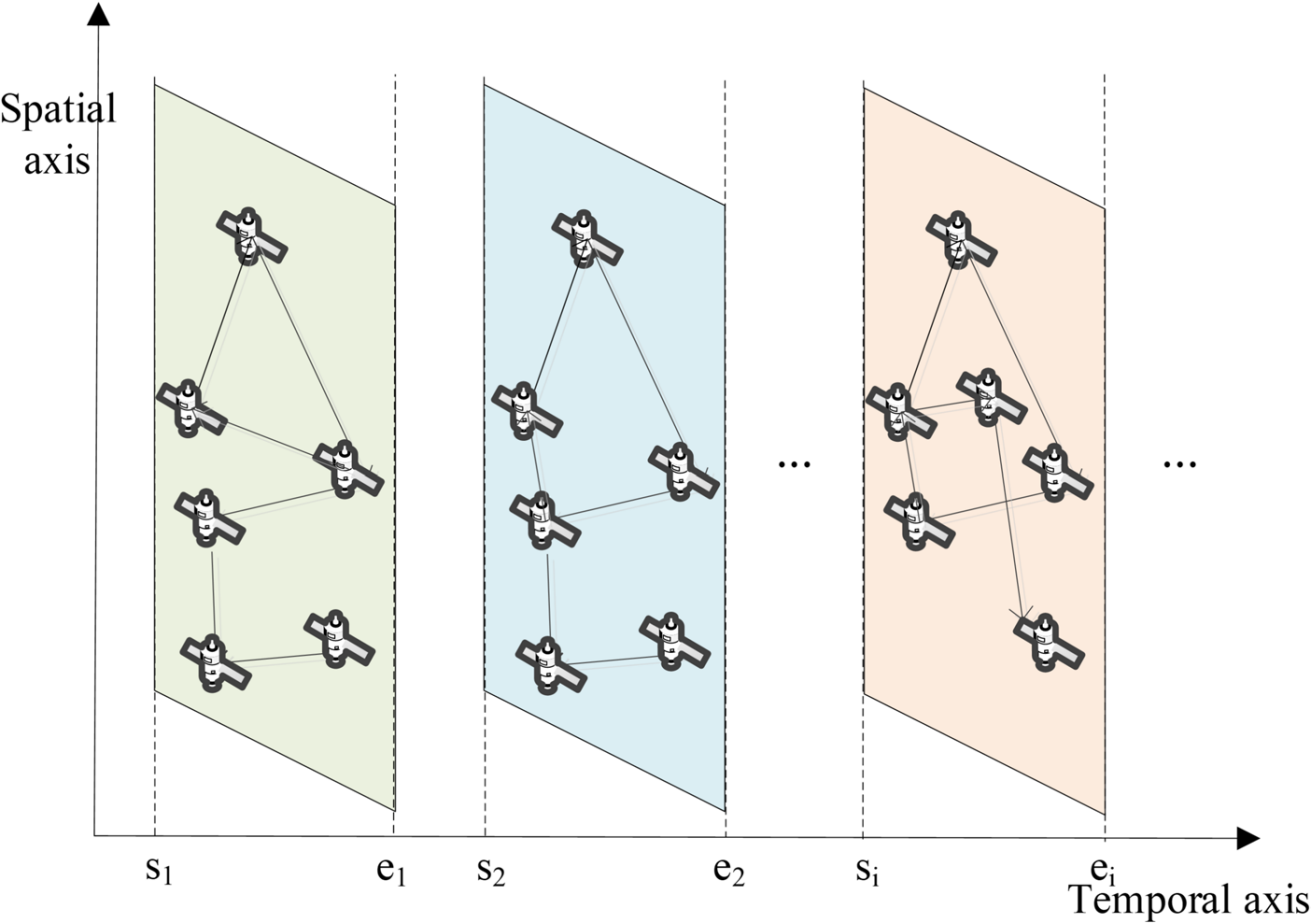
A dynamic topology analysis method based on time slicing.

Unlike terrestrial network models, satellite networks are dynamic and their topology undergoes periodic changes over time. To address this challenge, time slicing is employed to divide the satellite network into discrete time slots, as illustrated in Fig. 2. Within each time slot, the satellite network topology is treated as static, allowing task offloading to be performed more effectively. This approach significantly reduces the complexity of solving the task offloading problem in satellite networks.

### Resource model

Satellite resources consist of computing, storage, and communication resources. To address the task offloading problem within a single time slice in a satellite edge computing network, it is assumed that the end user is covered by *n* edge computing satellite (ECS) nodes during time slice *t*. The set of satellite nodes is represented as:

3 $$\begin{aligned} {ECS}_t=\left( V_1,V_2,\cdots ,V_n\right) . \end{aligned}$$

Each satellite node is defined by a five-tuple that represents its heterogeneous resources:

4 $$\begin{aligned} V_i=\left( ID,f_c,R_c,R_s,R_L\right) . \end{aligned}$$

Here, ID is the unique identification of the resource, $f_c$ denotes the computing speed of the satellite node, characterized by its CPU frequency, $R_c$ indicates the computing resource capacity of the satellite node, $R_s$ represents the storage resource capacity of the satellite node, and $R_L$ describes the connections between different nodes, which can be expressed as a matrix:

5 $$\begin{aligned} R_L^{n\bullet n}=\left[ \begin{matrix}e_{11}& \cdots & e_{1n}\\ \vdots & \ddots & \vdots \\ e_{n1}& \cdots & e_{nn}\\ \end{matrix}\right] . \end{aligned}$$

Where $e_{ij}$ is the link bandwidth resource between satellite node *i* and satellite node *j* in the current time slot. When $i=j, e_{ij}=0$.

### Task model

To effectively implement the task offloading strategy, it is essential to model large-scale task offloading requests in a reasonable manner^33^. Assume that a sequence of task requests is generated by *m* terminals, where each task is non-splitable:

6 $$\begin{aligned} Tasks=\left\{ T_1,T_2,\cdots ,T_m\right\} . \end{aligned}$$

Each task model is represented by a seven-tuple^34^:

7 $$\begin{aligned} T_i=\left\{ P,Data,T_C,T_S,T_L,T_{start},T_{dl}\right\} . \end{aligned}$$

Here, *P* represents the task priority, which is determined by the task type and is ranked as shown in Table 2. Data refers to the data size of the task. $T_C$ denotes the computational resources required for the task, expressed as the number of processing cycles needed by the satellite CPUs. $T_S$ represents the storage resources required for the task, where $T_L$ indicates the link resources needed. $T_{start}$ specifies the start time of the task, and $T_{dl}$ represents the maximum latency the task can tolerate.

**Table 2:** Task priority.

Priority	Task type
*P*5	Wartime tasks
*P*4	Conventional military tasks
*P*3	Large-scale factory tasks
*P*2	Large-scale school tasks
*P*1	Large-scale corporate tasks
*P*0	Personal tasks

### Computation offloading model

In task offloading decisions, the objective is to assign tasks to appropriate satellite nodes. Before selecting a satellite node, it is essential to consider the latency caused by task execution on each ECS node^35^. Due to the relatively short distance between LEO satellites and the ground, the propagation distance of tasks from the terminal to the satellite node is minimal, and the propagation speed is nearly equal to the speed of light. As a result, the propagation latency is negligible, and only computation latency and transmission latency are considered.

1. Computation latency
  Computation latency refers to the time required by a satellite to process a given task. The computation latency for satellite *j* to process task *i* is expressed as:
  8 $$\begin{aligned} T_{i,proc}^j=\frac{T_{C,i}}{f_{j,i}}+T_{i,que}. \end{aligned}$$
2. Transmission latency
  In this paper, we focus solely on the transmission latency of tasks from the terminal to the satellite node. The transmission delay for terminal *i* sending data to satellite *j* is denoted as:
  9 $$\begin{aligned} T_{i,trans}=\frac{{Data}_i}{r_{T_i,V_j}^{uplink}}. \end{aligned}$$
  Where $r_{T_i,V_j}^{uplink}$ is the transmission rate of the sub-channel assigned to task *i* by satellite *j*. Therefore, the total processing latency of task *i* is
  10 $$\begin{aligned} T_{i,total}=T_{i,proc}^j+T_{i,trans}^j. \end{aligned}$$

### Task offloading constraints

Assume that there are $Tasks=\left\{ T_1,T_2,\cdots ,T_m\right\}$ offloaded to satellite node *j* simultaneously.

1. Resource node constraints
  When offloading tasks, ECS nodes must have sufficient resources. For the terminal task request sequence defined by Equation (6), the following conditions must be satisfied for any satellite node *j*:
  11 $$\begin{aligned} \left\{ \begin{array}{cc} \sum _{i=1}^{m} T_C^i \le R_C^J & \\ \sum _{i=1}^{m} T_S^i \le R_S^J & \end{array} \right. \end{aligned}$$
  The total computational resources required to process all tasks on satellite node *j* must not exceed the node’s available computational capacity, and the total storage space occupied by all tasks must not surpass the node’s storage capacity.
2. Link Bandwidth Constraints
  The bandwidth required for transmissions over the link must not exceed the total available bandwidth of the link.
  12 $$\begin{aligned} \left\{ \sum _{i=1}^{m} T_L^i \le R_L^J \right. \end{aligned}$$
3. Response time constraints
  For any computational task *i*, the response time must not exceed its specified maximum allowable latency.
  13 $$\begin{aligned} T_{i,total}\le T_{dl}. \end{aligned}$$
4. Satellite visible time constraints
  Define the task decision time as ${ST}_i$. Within the time slice *t*, terminal tasks can only be offloaded to the ECS node currently covering the region. Once the time slice ends, the edge computing satellite cluster changes, rendering the current offloading decision invalid. This results in the failure of the task offloading process and requires a new decision to be made. Let the start and end times of the time slice be defined as $\left\{ {begin}_t,{end}_t\right\}$:
  14 $$\begin{aligned} {begin}_t\le {ST}_i\le {end}_t. \end{aligned}$$

### Multi-objective optimization function

The overall system resource utilization, defined as the weighted sum of computational resource utilization, storage resource utilization, and link resource utilization, is expressed as:

15 $$\begin{aligned} Rate_{total}=\alpha Rate_C+\beta Rate_S+\gamma Rate_L. \end{aligned}$$

Where $\alpha$, $\beta$, and $\gamma$ are the weights assigned to each type of resource,representing their respective influence on the overall resource utilization, with the condition that $\alpha +\beta +\gamma =1$. $Rate_C$, $Rate_S$, and $Rate_L$ denote the utilization rates of computational resources, storage resources, and link resources, respectively.

The load balancing degree, denoted as $r_{lb}$, is a system-level metric quantifying the resource utilization uniformity across n nodes in distributed systems, with a range within [0,1], where $r_{lb} \rightarrow 1$ indicates higher balance. The load balancing degree calculation is as follows:

16 $$\begin{aligned} r_{lb}= {\left\{ \begin{array}{ll} 1 & \text {if } \mu = 0 \\ \dfrac{\mu }{\mu + \delta } & \text {otherwise} \end{array}\right. } \end{aligned}$$

where $\mu$ and $\delta$ can be calculated as follows:

17 $$\begin{aligned} \mu = E_{rate}=\dfrac{1}{n}\sum _{i=1}^{n}Rate_{total}^i \ \end{aligned}$$

18 $$\begin{aligned} \delta = \sqrt{\frac{1}{n} \sum _{i=1}^{n} \left( {Rate}_{{total}}^{i} - \mu \right) ^{2}} \end{aligned}$$

when $\mu =0$ indicates that the system is in an idle state, with no active tasks being processed.

The goal of task offloading is to optimize the processing latency of all tasks, improve the task completion rate, and enhance resource utilization and load balancing within the satellite cluster, all while maintaining the quality of service for users. This approach ensures efficient resource allocation and prevents satellite nodes from becoming overloaded. The optimization problem is formulated as follows:

19 $$\begin{aligned} \left\{ \begin{array}{ll} {Min\ T}_{i,total} \\ {Max\ Rate}_{total} \\ {Max\ r}_{lb} \end{array} \right. \end{aligned}$$

To construct the reward function for multi-objective optimization, the task response latency is normalized as follows:

20 $$\begin{aligned} T_{i,total}^*=\frac{T_{i,total}}{T_{i,dl}-T_{i,start}}. \end{aligned}$$

Where $T_{i,dl}$ denotes the maximum processing latency of task *i* offloading to different satellite nodes, and $T_{i,start}$ is the start time of task *i* offloading. The normalized task latency $T_{i,total}^*\in \left[ 0,1\right]$.

Based on the constraints outlined in Equations (11)-(14), the multi-objective optimization function in this paper is defined as follows:

21 $$\begin{aligned} Reward=-\alpha T_{i,total}^*+\beta Rate_{total}+\gamma r_{lb}. \end{aligned}$$

$\alpha$, $\beta$, and $\gamma$ are weight coefficients, which can be set differently according to different types of application, and $\alpha +\beta +\gamma =1$. In this paper, the coefficients are set as: $\alpha$ = 0.4, $\beta$ = 0.3, $\gamma$ = 0.3.

## Task offloading decision algorithm based on K-D3QN

Reinforcement learning (RL) algorithms^36^ can rapidly provide offloading decisions to meet the high real-time requirements. Intelligent agents execute corresponding action strategies based on observations of the surrounding environment at different stages and optimize their actions using feedback from various environments^37^. These stochastic characteristics make RL particularly well-suited for satellite edge computing environments, where resources are constantly changing. In this paper, we propose a dynamic, multi-objective optimized offloading decision method based on an improved DQN algorithm, specifically utilizing K-D3QN for multi-objective optimization.

The optimization problem (19) is modeled as a constrained MDP, which is a tuple $<S, A, R>$, and the details of each element can be given as follows:

**State:** $S=\left\{ S_t|S_t=\left( S_{sec},S_{task},S_c\right) \right\}$, where each time slot t corresponds state $S_t, S_t\in S$. $S_{sec}$ represents the resource status of ECS, $S_{task}$ represents the task requirements of ECS and $S_c$ represents the channel status of the cluster.

**Action:** $A=\left\{ A_t|A_t={a_i|i\in {1,2,\ldots ,m}}\right\}$, where each time slot t the agent sends the offloading decision to the terminal. $a_i={1,2,\ldots ,n}$, when $a_i=0$, it indicates the offloading failure; otherwise, it indicates that the task *i* will be offloaded to the corresponding satellite node.

**Reward:** The reward function in this paper is related to the optimization objective, $r_{s(t)}^a=-Reward=-(-\alpha T_{i,total}^*+\beta Rate_{total}+\gamma r_{lb})$.

**Fig. 3:**
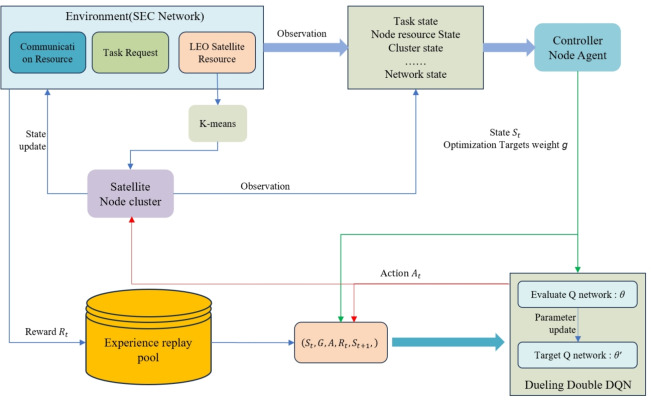
Task offloading model based on K-D3QN.

The model of dynamic multi-objective optimization offloading decision based on K-D3QN is illustrated in Fig. 3.

### K-means based clustering of satellite resources

The K-means algorithm is a clustering method that relies on Euclidean distance to measure similarity. The Euclidean distance is typically defined as follows:

22 $$\begin{aligned} d\left( x_i,c_i\right) =\sqrt{\sum _{j=1}^{m}\left( x_{ij}-c_{ij}\right) ^2}. \end{aligned}$$

Where $x_i$ is the sample object, $c_i$ is the *i*th cluster center, *m* is the feature dimension of the sample, and $x_{ij}$ and $c_{ij}$ are the *j*th feature values of the sample and cluster center.

In this paper, we utilize the K-means clustering algorithm to group edge satellite nodes based on their computing, storage, and bandwidth resources. Depending on the resource characteristics of ECS nodes, they are categorized into different clusters, including computational nodes, storage nodes, communication nodes, computational-storage nodes, storage-communication nodes, and computational-communication nodes. When a task offloading request arrives, the scheduler assigns the appropriate satellite cluster based on the task’s characteristics. This clustering approach significantly reduces the search space for task offloading decisions, thereby improving the algorithm’s efficiency and response time.

The resource clustering features of ECS are normalized as follows:

23 $$\begin{aligned} x_{stand}=\frac{x-x_{mean}}{\sigma }. \end{aligned}$$

Here, $x_{mean}$ represents the mean of the features and $\sigma$ denotes the standard deviation of the features. Based on these clustering features, the K-means algorithm is applied to group all ECS nodes into *k* satellite resource clusters. By utilizing the clustering algorithm, the action space for offloading decisions is transformed as follows:

24 $$\begin{aligned} \left( N+1\right) ^M\rightarrow \left( k+1\right) ^M. \end{aligned}$$

Where *N* represents the number of ECS nodes and *k* represents the number of clusters.

### Algorithm

D3QN integrates the features of Double DQN and Dueling DQN, significantly enhancing the efficiency of the DQN algorithm. To address the issue of Q-value overestimation in the DQN model, this paper improves the algorithm’s efficiency by incorporating DDQN. This approach refines the parameter update rule and estimates the maximum Q-value of the next state based on the parameters of the target network. The updated rule is as follows:

25 $$\begin{aligned} Y_t^{\textrm{DDQN}}=R_t+\lambda Q\left( S_{t+1},\ arg\underset{\text {a}}{max} Q\left( S_{t+1},a;\theta _t\right) ,\theta _t^-\right) . \end{aligned}$$

The loss function is:

26 $$\begin{aligned} L\left( \theta \right) =E\left[ \left( Y_t^{\textrm{DDQN}}-Q\left( s,a;\theta \right) \right) ^2\right] . \end{aligned}$$

According to the DDQN parameter update rule, the Q-value should not exceed the Q-value calculated using the target network. This approach mitigates the problem of Q-value overestimation, ensuring that the Q-value of actions is closer to their true value. As a result, the final offloading decision aligns more closely with the actual situation, and the optimal action receives higher reward values.

Additionally, instead of directly training the network to compute the Q-value, the fully connected layer of the DQN is enhanced by introducing two neural network outputs to derive two intermediate variables: V (state value) and A (action advantage). These variables are then combined to calculate the Q-value. The variable V represents the average expected Q-value for all task offloading actions in the current state, while A indicates the advantage of each specific offloading action. However, if the neural network trains V to a fixed value of 0, the distinction between Dueling DQN and standard DQN is lost. To address this, it is crucial to normalize the value of A across all actions, ensuring that the average value of action advantages is 0. The normalization process is as follows:

27 $$\begin{aligned} \bar{A}\left( s,a;\theta ,\alpha \right) =A\left( s,a;\theta ,\alpha \right) -\frac{1}{m}\sum _{j=1}^{m} A\left( s,a^\prime ;\theta ,\alpha \right) . \end{aligned}$$

Here, $\alpha$ and $\beta$ represent the parameters of the two neural networks in the fully connected layer, $\theta$ denotes the parameters of the convolutional layer, and *m* is the dimension of the action vector. The normalization process for the action advantage value of each action *a* involves subtracting the expected value of all actions in the current state from the dominance value of each action. This ensures that the A value of each action accurately reflects its relative superiority. Consequently, the Q-value of each action is calculated as the sum of the state value and the normalized action advantage value, as expressed by:

28 $$\begin{aligned} Q\left( s,a;\theta ,\alpha ,\beta \right) =V\left( s;\theta ,\beta \right) +\ \left( A\left( s,a;\theta ,\alpha \right) -\frac{1}{m}\sum _{j=1}^{m} A\left( s,a^\prime ;\theta ,\alpha \right) \right) . \end{aligned}$$

By integrating an optimization target weight at the input layer of the D3QN model and using a competitive network mechanism at the output layer to normalize both the state value and action advantage values, the Q-value for each action in the current state is calculated. Subsequently, the action with the highest Q-value is selected as the optimal decision for dynamic multi-objective optimization in the task offloading process. The details of K-D3QN are shown in Algorithm 1.

**Algorithm 1:**
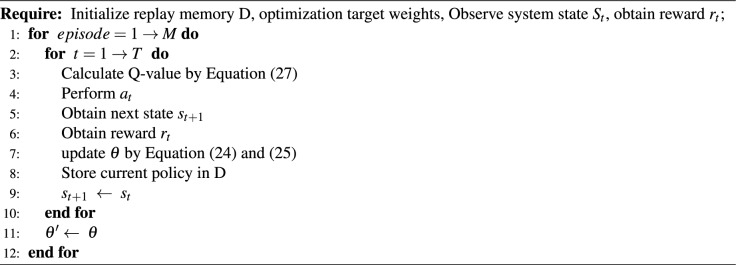
The K-D3QN Algorithm.

## Simulation and analysis

In order to verify the effectiveness of the proposed algorithm, we established a SEC scenario based on Satellite Tool Kit(STK), consists of LEO satellites, a ground station, and ground terminals. The important parameters are listed in Table 3.

**Table 3:** Parameter settings.

Parameter	Value
Number of LEOs	[50, 100]
Number of ground stations	1
Number of ground terminals	[100, 500]
time slot (s)	5
Computing capability of LEO satellite (Gcycles/s)	2
Storage capability of LEO satellite (GB)	10
Altitude of LEO satellite(KM)	780
Data size of task $T_i$(MB)	[1, 10]
computation size of task $T_i$(Kcycles/bit)	[200, 500]
Weight for optimization target *g*	(0.4, 0.3, 0.3)

To validate the performance of K-D3QN, we compare the proposed algorithm with benchmark algorithms, including the DQN algorithm and the particle ant colony optimization (ACO) algorithm, focusing on task response time, task offloading success rate and load balancing degree. The descriptions of the these algorithm are as follows:

- DQN: DQN is a reinforcement learning algorithm that uses a deep neural network to approximate the Q-value function, enabling agents to make optimal decisions in complex environments.
- ACO: ACO is a meta-heuristic algorithm that simulates the foraging behavior of ants to solve optimization problems.

**Fig. 4:**
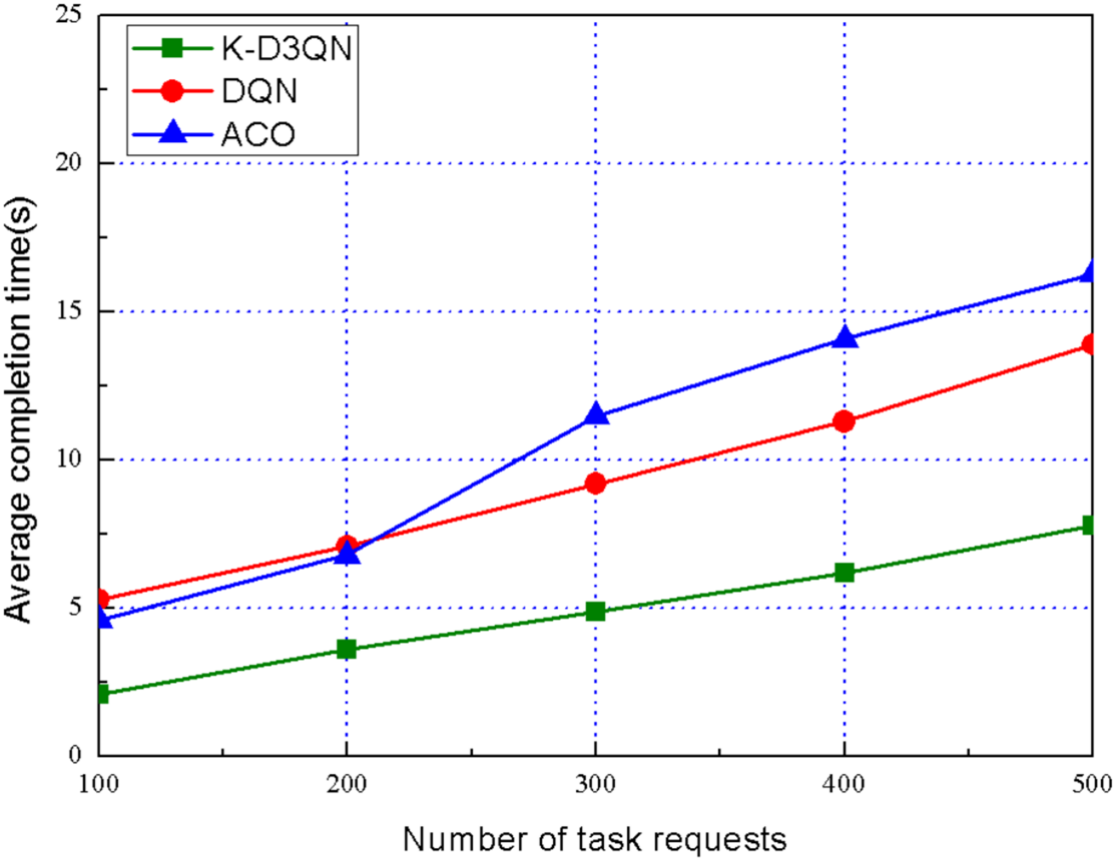
Average completion time of different task requests.

**Fig. 5:**
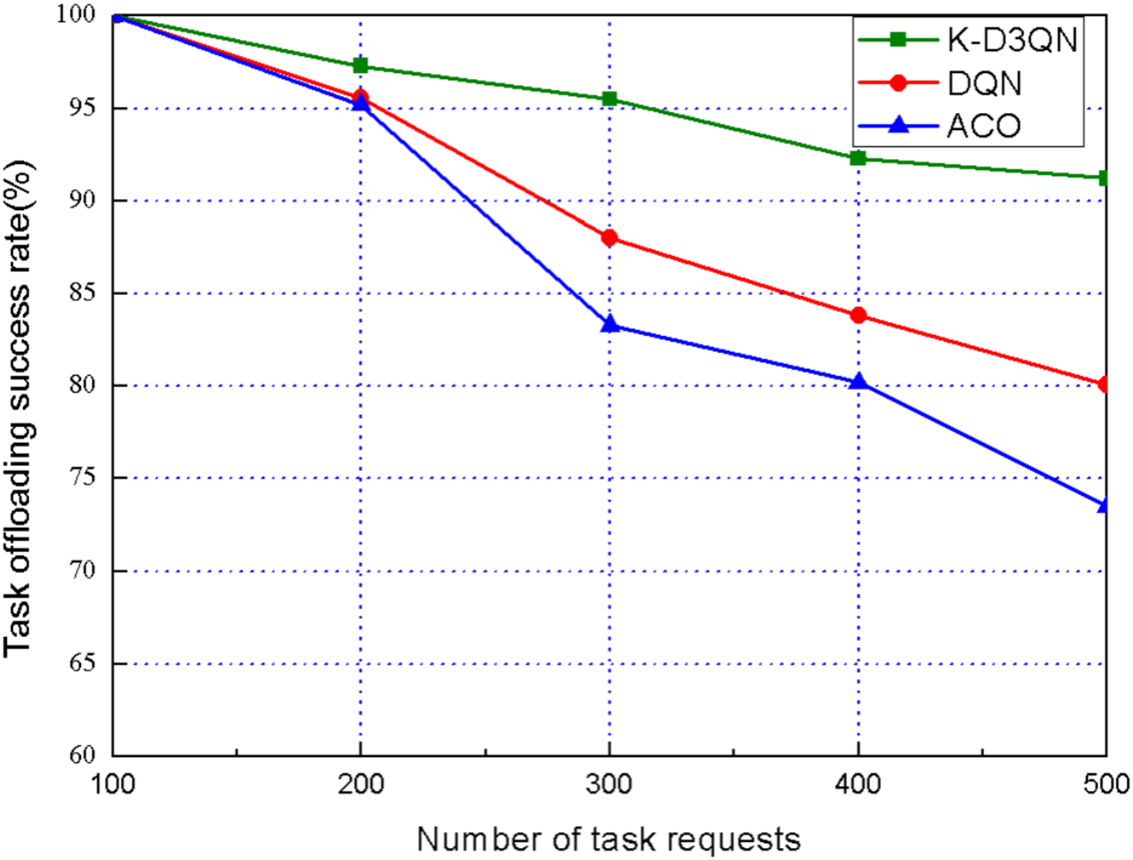
Task offloading success rate of different task requests.

**Fig. 6:**
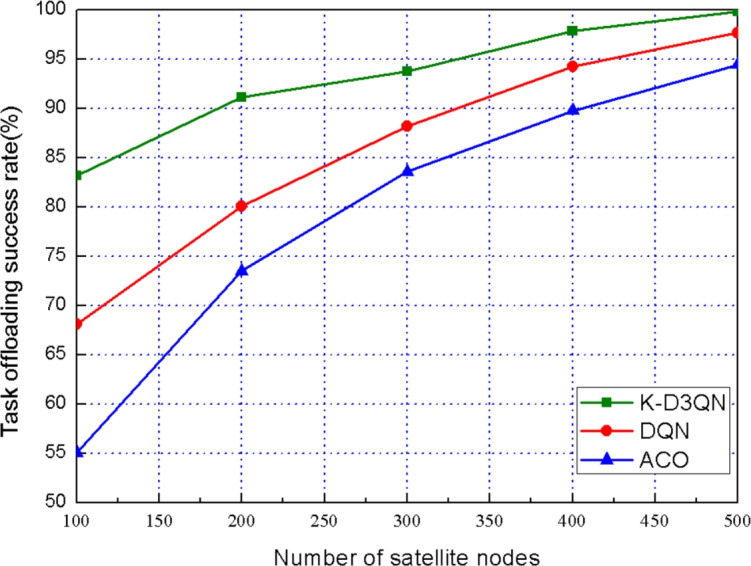
Task offloading success rate of different satellite nodes.

**Fig. 7:**
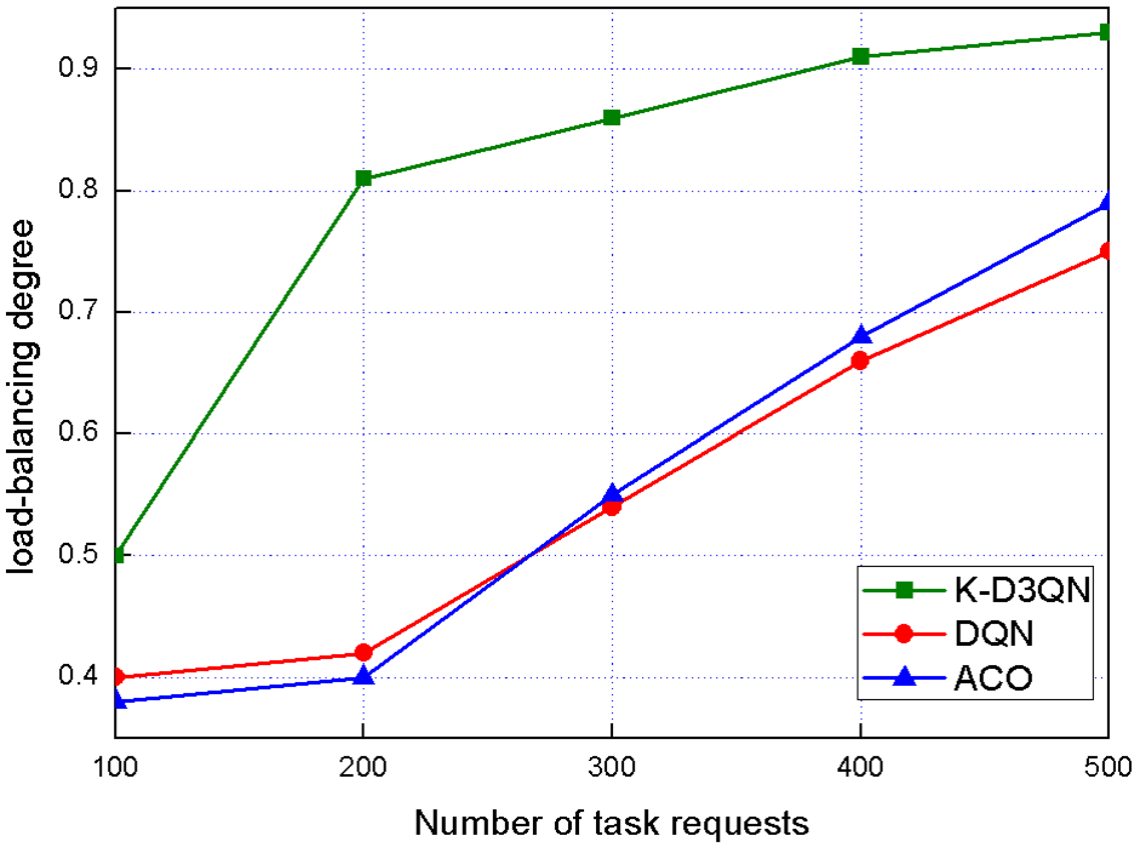
Load-balancing degree of different task requests.

Figure 4 compares the average completion time of different algorithms under varying task offloading requests. In this experiment, the number of satellite nodes is set to 200, and the simulation is repeated 10 times to ensure the reliability of the results. With the increase of the number of task requests, the average completion time increasing. It can be seen that the performance of K-D3QN is better than DQN and ACO. Specifically, when the number of task requests exceeds 400, the average completion time of the K-D3QN algorithm remains relatively stable, while the completion time of the baseline algorithms rises sharply. This stability is attributed to the dynamic multi-objective optimization employed by the K-D3QN algorithm, which effectively balances task latency, system resource utilization, and load-balancing.

To verify the success rate of task offloading, the performance of the algorithms was evaluated in the satellite edge computing scenario. The analysis was conducted with varying numbers of tasks, while the number of satellite nodes was fixed at 200. The experimental results are illustrated in Fig. 5. It can be seen from Fig. 5, when the number of task requests is 100, all algorithms achieve a 100% task offloading success rate. However, as the number of task requests increases, the task offloading success rate of all algorithms decreases. The experimental comparison clearly demonstrates that the K-D3QN algorithm consistently outperforms the DQN algorithm and the ACO algorithm in terms of task offloading success rate, regardless of the number of task requests. This highlights the robustness and efficiency of the K-D3QN algorithm in handling varying workloads in satellite edge computing scenarios.

To further evaluate the impact of task offloading success rates across different satellite nodes, we compared the performance of the three algorithms with the number of task requests fixed at 500. The results, shown in Fig. 6, indicate that the success rate of task offloading increases as the number of satellite nodes increases. Moreover, the K-D3QN algorithm consistently outperforms both the DQN algorithm and the ACO algorithm in terms of task offloading success rate.

Figure 7 illustrates the system’s load-balancing degree as the number of task requests varies from 100 to 500, with the number of LEO satellites fixed at 200. When the number of task requests is below 200, the load-balancing degree is relatively low for all algorithms. This is because the number of tasks is smaller than the number of satellite nodes, leading to uneven task distribution. However, when the number of task requests exceeds the number of satellite nodes, the K-D3QN algorithm demonstrates a higher load-balancing degree. Its optimization objective takes into account task latency, system resource utilization, and load-balancing degree, enabling more efficient task distribution and better resource utilization. In comparison, the DQN and ACO algorithms show relatively lower load-balancing degrees in this scenario.

## Conclusions

In this paper, we studied the task offloading algorithm for satellite edge computing to minimize the latency, improve the resource utilization and load-balancing degree. we constructed the system resource model and task model based on the LEO edge computing network architecture and defined the system constraints and optimization objectives. Then, we formulated the problem as a Markov Decision Process (MDP) and proposed the K-D3QN algorithm to enhance the success rate of terminal task offloading and reduce task response time. Finally, to validate the effectiveness of the proposed method, a simulation environment was constructed using STK. The experimental results show that, compared to the baseline methods, the proposed approach significantly reduces task completion time, enhances the task offload success rate and load-balancing degree. In the future, we will develop a network topology prediction model based on the highly dynamic characteristic of satellite networks to dynamically predict topology changes, and study the multi-objective optimization offloading algorithm.

## Data Availability

The datasets used and/or analyzed during the current study available from the corresponding author on reasonable request.
